# Retention of the virus-derived sequences in the nuclear genome of grapevine as a potential pathway to virus resistance

**DOI:** 10.1186/1745-6150-4-21

**Published:** 2009-06-26

**Authors:** Christophe Bertsch, Monique Beuve, Valerian V Dolja, Marion Wirth, Frédérique Pelsy, Etienne Herrbach, Olivier Lemaire

**Affiliations:** 1 Université de Haute-Alsace, Laboratoire Vigne Biotechnologie et Environement EA 3991, 33 rue de Herrlisheim 68000 Colmar, France; 2 INRA, UMR 1131 Santé de la Vigne et Qualité du Vin, 28 rue de Herrlisheim, BP20507, 68000 Colmar, France; 3 Université de Strasbourg, UMR 1131, F-67000 Strasbourg, France; 4 Department of Botany and Plant Pathology and Center for Genome Research and Biocomputing, Oregon State University, Corvallis, OR 97331, USA

## Abstract

**Background:**

Previous studies have revealed a wide-spread occurence of the partial and complete genomes of the reverse-transcribing pararetroviruses in the nuclear genomes of herbaceous plants. Although the absence of the virus-encoded integrases attests to the random and incidental incorporation of the viral sequences, their presence could have functional implications for the virus-host interactions.

**Hypothesis:**

Analyses of two nuclear genomes of grapevine revealed multiple events of horizontal gene transfer from pararetroviruses. The ~200–800 bp inserts that corresponded to partial ORFs encoding reverse transcriptase apparently derived from unknown or extinct caulimoviruses and tungroviruses, were found in 11 grapevine chromosomes. In contrast to the previous reports, no reliable cases of the inserts derived from the positive-strand RNA viruses were found. Because grapevine is known to be infected by the diverse positive-strand RNA viruses, but not pararetroviruses, we hypothesize that pararetroviral inserts have conferred host resistance to these viruses. Furthermore, we propose that such resistance involves RNA interference-related mechanisms acting via small RNA-mediated methylation of pararetroviral DNAs and/or via degradation of the viral mRNAs.

**Conclusion:**

The pararetroviral sequences in plant genomes may be maintained due to the benefits of virus resistance to this class of viruses conferred by their presence. Such resistance could be particularly significant for the woody plants that must withstand years- to centuries-long virus assault. Experimental research into the RNA interference pathways involving the integrated pararetroviral inserts is required to test this hypothesis.

**Reviewers:**

This article was reviewed by Arcady R. Mushegian, I. King Jordan, and Eugene V. Koonin.

## Background

A recent concept of the Virus World based on the comparative genomics traces the origins of 'viral hallmark genes' that are broadly distributed among RNA, DNA, and retroid viruses and parasitic elements to the precellular genetic systems [1]. At the same time, this concept emphasizes a tight connection between evolution of viruses and cells that involves numerous events of horizontal gene transfer, or HGT [2,3]. Such bidirectional gene flow between viruses and cells is evident from the presence of the homologs of cellular genes in viral genomes and presence of proviruses and virus-derived genes in prokaryotic and eukaryotic genomes [4,5]. Some transfers of viral genes to host organisms appear to be very ancient, whereas others are relatively recent. The sizes of virus-derived inserts range from short stretches of the bacteriophage genomes used for antiviral defense [6] to the entire viral genomes capable of resurrecting infectious viruses, as in the case of virion DNA-containing, reverse-transcribing, plant pararetroviruses [7-9]. Unlike animal RNA-containing retroviruses that use genome integration as a part of their infection cycle, plant pararetroviruses lack integrase or other means for active host genome invasion. This, and the normal exclusion of viruses from the plant germ cells, reduce the likelihood of genome integration and suggest that a heritable maintenance of pararetroviral sequences has potential benefits for the host plants [10]. Whereas the presence of the viral inserts derived from DNA-containing viruses in plant genomes is well established [7,11], the case for such inserts from positive-strand RNA viruses remained a matter of debate.

In the last few years, sequencing of the several plant genomes provided an opportunity to search for the virus-specific sequences homologous to both DNA- and RNA-containing viruses. Here, we analyzed two annotated genomes of the grapevine (*Vitis vinifera*), Pinot Noir-derived line PN40024 [12] and Pinot Noir clone ENTAV 115 [13], for the presence of viral sequences. In addition, we conducted a similar, although more limited analysis of the poplar (*Populus trichocarpa*) genome [14]. The obtained data, combined with the existing information on the extant viruses of grapevine and poplar, are compatible with the hypothesis according to which stochastic acquisition of the sequences derived from the pararetroviruses, but not RNA viruses, made the pararetroviruses capable of infecting these woody plants exceptionally rare if not extinct.

## Presentation of the hypothesis

**Figure 1:**
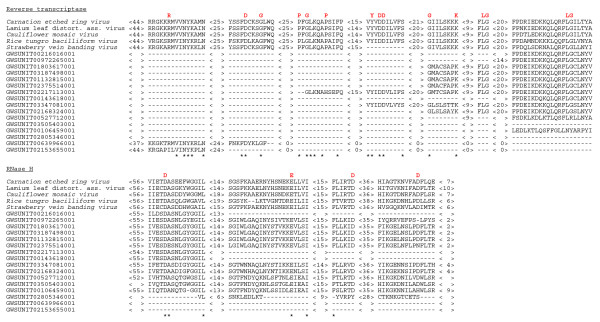
**Multiple alignment of the amino acids sequences corresponding to the reverse transcriptase and RNase H domains of *Carnation etched ring virus *(NP_612577), *Lamium leaf distortion associated virus *(YP_001931961.1), *Cauliflower mosaic virus *(AAD37341), *Rice tungro bacilliform virus *(FAA00012.1), *Strawberry vein banding virus *(NP_043933.1) and the homologous inserts from grapevine**. The alignments were obtained using the Clustal W2 program. The numbers indicate the lengths of amino acid sequences between the conserved motifs. The invariant amino acid residues are highlighted in red, whereas the conserved residues are marked by asterisks.

The 913 apparent sequence matches with the putative viral nucleotide sequences were identified using the Grape Genome Browser . Blast analysis of these virus-related inserts revealed sixteen potential ORFs that exhibited the highest, 30–60% identity to the protein sequences of five distinct pararetroviruses of the family *Caulimoviridae*, genera *Tungrovirus *and *Caulimovirus *(Table 1) [15]. Each of these ORFs was incomplete relative to the homologus pararetroviral ORFs. Further comparisons of the viral and grapevine genome-derived protein sequences using ClustalW2  identified several amino acid motifs conserved in the pararetroviral reverse transcriptase (RT) and RNase H domains (Fig. 1). The inserts varied in lengths from ~200 to ~800 nucleotides and were scattered in an apparently random fashion among 11 of the 19 grapevine chromosomes (Additional file 1). Some of the remaining sequence matches corresponded to the other pararetroviral ORFs, most notably, those encoding the viral movement proteins. These inserts were not linked to the partial RT-encoding ORFs, exhibited lower similarity, and were not analyzed in more detail.

**Table 1:** Virus-derived sequences in the the grapevine genome.

**Virus family, genus, and species**	**Related gene**	**% a.a. identity**	**Accession code; chrom. number and position; Genoscope 40024**	**Contig and position; IASMA 115 ENTAV**
***Caulimoviridae, Tungovirus***				
*Rice tungro bacilliform virus*	hyp. prot. (RT-like)	60%	GWSUNIT02805346001 chr1 random:120,261,152..120,261,368 (217 bp)	VV78X109220.8 (5,691..5,480)
*Rice tungro bacilliform virus*	hyp. prot. (RT-like)	41%	GWSUNIT02217113001 chr3:8,822,689..8,823,246 (558 pb)	VV78X056749.7 (50,560..50,003)
*Rice tungro bacilliform virus*	hyp. prot. (RT-like)	47%	GWSUNIT00527712001 chr3:9,805,215..9,805,809 (595 pb)	VV78X138621.5 (894..1,481)
*Rice tungro bacilliform virus*	hyp. prot. (RT-like)	47%	GWSUNIT03505403001 chr5:18,556,193..18,556,564 (372 pb)	VV78X212046.4 (3,752..4,123)
*Rice tungro bacilliform virus*	hyp. prot. (RT-like)	41%	GWSUNIT00106459001 chr16:4,304,137..4,304,751 (615 pb)	VV78X023914.6 (2,142..2,756)

***Caulimoviridae, Caulimovirus***				
*Cauliflower mosaic virus*	ORFV (RT- like)	42%	GWSUNIT00143618001 chr2:8,872,185..8,872,457 (273 pb)	VV78X033617.59 (6,979..6,711)
*Strawberry vein banding virus*	ORFV (hyp. prot. gp5, RT-like)	59%	GWSUNIT00639966001 chr6:12,644,211..12,644,470 (259 bp)	VV78X129432.8 (1757..1498)
*Strawberry vein banding virus*	ORFV (hyp. prot. gp5, RT-like)	52%	GWSUNIT02153655001 chr8:4,148,212..4,148,460 (249 pb)	VV78X220330.2 (536..289)
Lamium leaf distortion associated virus	RT	40%	GWSUNIT03347081001 chr1 random:117,898,214..117,899,037 (824 pb)	VV78X277334.5 (2,278..1,453)
Lamium leaf distortion associated virus	RT	41%	GWSUNIT02168324001 chr8:6,149,976..6,150,701 (725 bp)	VV78X035320.7 (6,816..6,090)
*Carnation etched ring virus*	ORFV (RT- like)	42%	GWSUNIT00216016001 chr2:16,370,519..16,370,830 (312 pb)	VV79X003991.5 (14,124..14,435)
*Carnation etched ring virus*	ORFV (RT- like)	30%	GWSUNIT02375514001 chr4:13,714,314..13,715,033 (720 pb)	VV78X034163.2 (1530..2244)
*Carnation etched ring virus*	ORFV (RT- like)	34%	GWSUNIT01803617001 chr5:2,240,705..2,241,424 (720 pb)	VV78X065575.6 (4,015..4,734)
*Carnation etched ring virus*	ORFV (RT- like)	39%	GWSUNIT03187498001 chr10:4,303,075..4,303,794 (720 pb)	VV79X003991.5 (14,004..14,723)
*Carnation etched ring virus*	ORFV (RT- like)	37%	GWSUNIT00972265001 chr11:11,149,476..11,150,121 (646 pb)	VV78X111735.9 (2,609..1,964)
*Carnation etched ring virus*	ORFV (RT- like)	40%	GWSUNIT01132815001 chr17:2,076,764..2,077,483 (720 pb)	VV78X105441.6 (1,428..710)

Classification, related gene, protein level identity, and localization of the different putative phytovirus sequences integrated in the Pinot Noir-derived line PN40024 and Pinot Noir ENTAV 115 genome.

(hyp: hypothetic; prot: protein; RT: transcriptase reverse; chrom., chromosome)

At least eight ORF fragments encoding pararetroviral RTs were readily identified in the poplar genome. These ORFs showed 42–66% identity levels to six distinct tungro- and caulimoviruses (not shown). Because we used a strict cut-off value of 30% protein sequence identity to known pararetroviruses in order to exclude retroelements, the actual number of the pararetrovirus-derived inserts in the grapevine and poplar genomes is likely to be much higher than 16 and 8, respectively, perhaps, by an order of magnitude or more.

**Figure 2:**
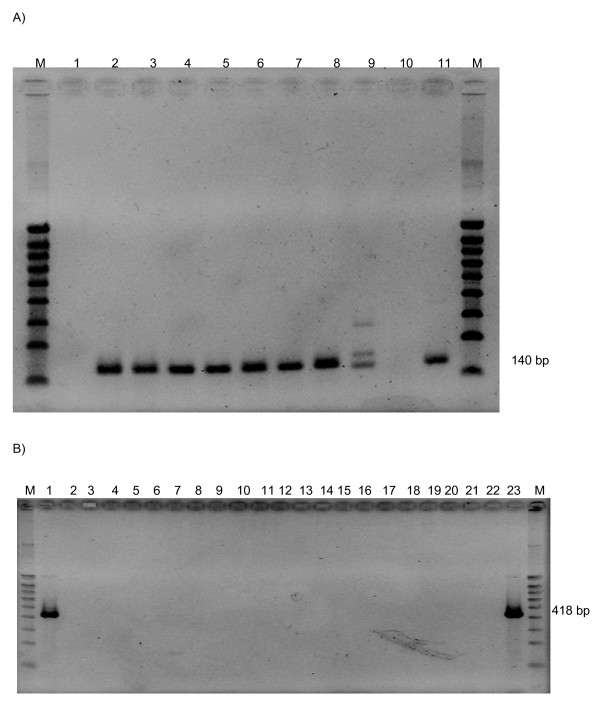
**(A) RT-PCR product of putative GLRaV-8-related DNA sequence in different grapevines genomes**. Agarose gel electrophoretic analysis of mRNA expression by RT-PCR with specific GLRaV-8 primers from DNA of different *Vitaceae*. *Vitis vinifera *PN40024 (lane 2), *V. vinifera *subsp. *sylvestris *(lane 3), *V. aestivalis *(lane 4), *V. mustangensis *(lane 5), *V. coignetiae *'Ishikari' (lane 6), *V. rupestris *(lane 7), *V. davidii *(lane 8), *Ampelopsis japonica *(lane 9), *Parthenocissus quinquefolia *(lane 10), *V. rotundifolia *(lane 11), negative control: water (lane1), 100 bp ladder (lane M). **(B) **PCR detection of the DNA sequence homolgous to PVY CP on the different *Vitaceae *accessions failed. Agarose gel electrophoretic analysis of DNA fragments amplified by PCR, with specific PVY primers, from DNA of different *Vitaceae*. *Vitis vinifera *PN40024 (lane 2), *V. vinifera *'Gamay' (lane 3), *V. vinifera *'Gouais' (lane 4), *V. vinifera *subsp. *sylvestris *(lane 5), *V. aestivalis *(lane 6), *V. berlandieri *(lane 7), *V. mustangensis *(lane 8), *V. coignetiae *'Ishikari' (lane 9), *V. rupestris *(lane 10), *V. davidii *(lane 11), *Ampelopsis japonica *(lane 11), *A. aconitifolia *(lane 12), *A. cordata *(lane 13), *A. heterophylla *(lane 14), *A. pedonculata *(lane 15) *Parthenocissus quinquefolia *(lane 16), *V. rotundifolia *'Carlos' (lane 17), *V. rotundifolia *'Dulcet' (lane 18), *V. rotundifolia *'Regale'(lane 19), *V. rotundifolia *'Y × C' (lane 20), positive control: PVY plasmid (lanes 1 and 23), negative control: water (lane 22), 100 bp ladder (lane M).

Intriguingly, we also revealed the presence of two short inserts that were reported to originate from the positive-strand RNA closteroviruses [16], *Grapevine leafroll-associated virus-1 *(GLRaV-1) [17] and *Grapevine leafroll-associated virus-8 *(GLRaV-8) [18]. Although the latter short sequence was claimed to belong to a capsid protein gene of a novel closterovirus, GLRaV-8 (Monis, 2000), BLASTP search showed no significant similarity to any viral sequences in the database. Therefore, this sequence is likely of the non-viral origin, and is a part of grapevine genome proper. This conclusion was further supported by the RT-PCR analysis which demonstrated the presence of this insert and its transcription in the multiple *Vitaceae *species from North America and Asia except in *Parthenocissus quinquefolia *and in *Ampelopsis japonica *(no amplification for *P. quinquefolia *and nonspecific amplification for *A. japonica*; all PCR fragments of 140 bp were sequenced, see Fig. 2A). Surprisingly, the insert identical to this misidentified sequence was also identified in the genome of the grapevine mitochondrion [19].

The case of apparent insert derived from a partially sequenced GLRaV-1 Hsp70 homolog (Hsp70h) (AAK38612; direct submission) is slightly more complex. Although most of this annotated sequence indeed belongs to GLRaV-1 Hsp70h (independently sequenced by Fazeli and Rezaian, 2000 [17]), the 18 codon-long stretch present in the grapevine genome shows only 5-residue overlap with the actual viral protein. Therefore, the deposited AAK38612 sequence resulted likely from a cloning artefact that yielded a chimeric ORF with the C-terminal part obtained via reverse transcription of the non-viral, grapevine genome-derived transcript.

Because it was reported that the genomes of several grapevine varieties contain inserts derived from the positive-strand *Potato virus Y *(PVY, *Potyvirus*) [20], we specifically investigated this issue, and found no evidence for the presence of such inserts in either ENTAV 115 or PN40024 genomes. Furthermore, we have performed PCR analyses using PVY-specific primers and DNAs isolated from 19 species and varieties of grapevine including Gamay also used by Tanne and Sela [20]. Because no detectable PCR products were obtained (Fig. 2B), we concluded that there are no PVY-specific sequences inserted within the grapevine genome. Thus, the origin of the sequences reportedly derived from the positive-strand RNA closteroviruses and potyviruses seems to be exceedingly clear: human errors or misinterpretation. This outcome draws attention to the need in a more rigorous assessment of the published data and, especially, sequence annotations.

What are the origins and significance of the pararetroviral inserts in the grapevine genome? Integration via non-homologous recombination involving DNAs of the unknown or extinct, grapevine-infecting, viruses appears to be a likely scenario. Since the genetic cycle of these viruses involves formation of the RNA/DNA and ssDNA/dsDNA intermediates, recombination, perhaps aided by the host DNA repair machinery, seems to be a distinct possibility. The apparent lack of the complete viral ORFs or full-size genomes points to the incidental and aberrant nature of the insertion process, a conjecture compatible with the plant pararetroviral genetic cycle that does not require chromosome integration [21]. It seems plausible that stochastic transcription of the integrated viral sequences could generate virus-specific small interfering RNAs conferring an antiviral RNA interference response to the host plant [22].

Due to high cash value of the table and, especially, wine grapes, viral diseases of grapevine are a subject of thorough investigation by many labs worldwide. Dozens of viruses associated with these diseases were identified and characterized during several decades of the research. Conspicuously, every single virus found in grapevine belongs to the class of positive-strand RNA viruses with the broad representation of the families of the filamentous (*Flexiviridae *and *Closteroviridae*) and icosahedral (*Comoviridae*, *Tymoviridae*, and, occasionally, *Bromoviridae*) viruses [23]. A very recent study using deep sequencing of the transcriptome derived from the virus-affected vines resulted in identification of ~20 diverse viruses including a novel virus [24]; without a single exception, all these viruses were positive-strand RNA viruses from the families listed above. Although the viral diseases of poplar are explored to a much lesser extent, a few known poplar viruses also possess positive-strand RNA genomes [25].

Taken together, these considerations can be interpreted to mean that there are hardly any extant pararetroviruses capable of infecting, at least, the cultivated grapevine varieties. If so, it stands to reason that the decidedly pararetroviral inserts present in the grapevine genome were derived from the currently extinct, grapevine-specific pararetroviruses. Furthermore, given the broad occurrence of pararetroviruses in herbaceous monocot and dicot plants, it seems plausible that the woody plants such as grapevine and poplar took advantage of accumulation of pararetroviral inserts and adopted them to establish virus resistance. Perhaps, the most obvious way to do so would be to use the nuclear, pararetroviral inserts-derived, transcripts, to generate small RNA-effectors of the RNA interference pathways. Pararetroviruses are targets of at least two of these pathways acting via siRNA-mediated degradation of the viral mRNAs, and via small RNA-guided methylational inactivation of either episomal, or integrated viral DNA genomes [26,27].

It also seems possible that the endogenous virus sequence-dependent resistance mechanisms can operate in herbaceous plants, as was proposed for petunia (a dicot), and rice (a monocot) [28,29]. In contrast to grapevine, however, in these cases, plants exhibited either complete, or partial susceptibility to exogeneous or endogeneous (petunia) pararetrovirus challenges. Because one of the consequences of very long life spans of the woody plants is the dramatic increase in the exposure to viruses, it is possible that woody plants have evolved more robust RNAi defense responses than short-lived herbaceous plants.

Another interesting aspect of the grapevine-virus interactions is a preponderance of the positive-strand viruses in this host plant that resonates with the absence of the sequences derived from these viruses in the grapevine genome. One hypothesis explaining this correlation is that the lack of DNA phase in the genetic cycles of positive-strand RNA viruses dramatically reduces the chances of insertion of viral sequences into the host genome. The only feasible way to achieve such incorporation is reverse transcription of viral RNAs with the aid of either endogenous (retrotransposon-derived) RT, or RT expressed by a co-infecting retroid virus. Although theoretically possible, this pathway appears to be extremely inefficient. In fact, the only well-documented instance of an RNA virus-derived insertion in a nuclear genome via illegitimate recombination with retrotransposon was described in the mouse cell culture, but no evidence of the presence of such inserts in mouse genome was obtained [30]. Arguably, inability of RNA viruses to invade host genome may be considered as an evolutionary advantage that helps RNA viruses to evade pre-formed RNAi host defense response.

## Testing the hypothesis

Occurrence of the multiple pararetroviral inserts in the grapevine genome contrasted by the lack of known grapevine-infecting pararetroviruses represents an interesting evolutionary case where a host appears to win the arms race with an entire class of the retroid DNA viruses. We hypothesize that this outcome involved a recruitment of the byproducts of virus infections, the genome-incorporated viral sequences, by the host, to acquire virus resistance. As a result, grapevine pararetroviruses appear to be driven to a virtual extinction. It remains to be seen how common this phenomenon is among woody plants.

There are two research avenues that have a potential to either prove or refute our hypothesis. One is a census of all viruses in grapevine and other woody plants, now feasible with application of the metagenomics approaches. Another is investigation of the tree-specific RNAi mechanisms, especially those associated with DNA and, perhaps, RNA methylation. A role of the latter in viral infections was recently proposed on the basis of RNA repair activity of the AlkB domains present in a subset of the positive-strand RNA viruses that infect woody plants [31].

## Implications of the hypothesis

It will be important to see, if, indeed, some of the long-living woody plants have evolved a more potent, and, perhaps, more elaborate RNAi mechanisms to resist viral diseases than those present in herbaceous plants. If this were the case, such mechanisms can provide very useful means for improving virus resistance of the multitude of economically important herbaceous plants.

In addition, here we sorted out a controversial issue of the presence of RNA viral sequences in the grapevine genome and refuted the evidence for the existence of GLRaV-8. Taken together, these data have substantial implications for the identification and control of the grapevine viruses.

## Abbreviations

bp: base pair; DNA: deoxynuclei acid; ENTAV: établissement national technique pour l'amélioration de la viticulture; GLRaV-1: Grapevine leafroll-associated virus-1; GLRaV-8: Grapevine leafroll-associated virus-8; HGT: horizontal gene transfer; mRNA: messenger ribonucleic acid; ORF: open reading frame; PVY:Potato virus Y; RNA: ribonucleic acid; RNAi: ribonucleic acid interference; RT: reverse transcriptase; RT-PCR: reverse transcriptase-polymerase chain reaction; siRNA: short interfering ribonucleic acid.

## Competing interests

The authors declare that they have no competing interests.

## Authors' contributions

CB has done most of the bioinformatics work. MB and MM have conducted the experiments. CB, VVD, FP, EH and OL analyzed the data. OL has done general coordination and supervision. VVD and CB have written the manuscript.

## Reviewers' comments

### Reviewer's report 1

Dr. Arcady R. Mushegian (Stowers Institute for Medical Research, Kansas City, MO, USA)

*Reviewer comments:*

1. I agree that there seems to be no evidence of complete virus genomes integrated into Vitis genome, but what is the evidence that none of the virus reverse transcriptase-related inserts encode a complete ORF? Also, I have searched the NR protein database with the movement protein sequences (ORF1 in caulimoviruses) and can see many predicted proteins in Vitis – perhaps some of them may be expressed too?

2. The authors used 30% identity cutoff to distinguish between pararetrovirus-like sequences and retrotransposons that also have reverse transcriptases (and often gag/ORF4-like sequences in addition). Perhaps using aforementioned movement proteins as a marker would be more specific. It gives me an impression of a much high copy number of such inserts in grapevine compared to other completely sequenced genomes. A putative selective advantage, i.e., harnessing the spuriously transcribed inserts to protect from viruses, may perhaps explain the persistence of some inserts but not necessarily this high copy number. Can the accumulation of virus-like inserts in Vitis be a consequence of perennial lifestyle and clonal propagation (non-integrated viruses are typically excluded from seeds, limiting exposure in annuals)? I searched the whole-genome assembly of poplar P. trichocarpa for sequences related to caulimovirus movement proteins, and there appear to be two dozen matches at least, which seems compatible with this hypothesis. Discuss?

Minor: Ln 69–70 "unknown" includes "extinct" in this context, doesn't it?

*Authors' response:*

The manuscript was largely rewritten in response to these very useful comments with an emphasis on the potential selective advantage of the pararetroviral inserts for the perennial plants. The final variant of the work included a preliminary survey of the viral inserts not only in grapevine, but also in poplar genome. In accord with the reviewer's proposal, we have also assessed the presence of the pararetroviral movement protein gene-related inserts. As to the terms 'unknown' versus 'extinct', the former means extant yet unidentified viruses, whereas the latter applies to the viruses that no longer exist as the functional, infectious entities.

*Reviewer comments:*

1. Ln 38 in Abstract: change "active viral integration mechanisms" to "virus-encoded integrases".

2. Ln 41. Delete "Bioinformatics"?

3. Ln 44. Change "caulimo-" to "caulimoviruses". Can we confidently say that these sequences are not from other groups of caulimoviridae?

Ln 73 "DNA-containing" is a bit ambiguous, change to "virion DNA?"

Ln 89 change to "made the pararetroviruses.....woody plants exceptionally rare if not extinct".

Ln 92 and elsewhere: change "homology hits" and "hits" to "sequence matches" or "matches".

Lns 93–94 "hits with the putative viral nucleotide sequences" vs. "at the protein level": what was compared to what – details?

Lns 99–101: movement proteins are usually encoded on the same (35S) transcript as reverse transcriptases, so perhaps that fact that the two classes of matches are found separately is another indication that essentially random fragments of viral mRNA are incorporated into essentially random genomic locations (cf. Lns 144–146)? Also, change "less significant" to "lower" and delete "therefore".

Ln 104: explain the significance of the 30% identity cutoff: was it used to exclude retroelements, and how do we know this has been accomplished?

Ln 108: change "apparently derived" to "that were reported to originate"

Ln. 112–113 and Ln 125: delete "(not shown)".

Delete Ln 120.

Ln 136: delete "exceedingly"?

Ln 139: put "potential" in front of "origins" or, better, delet the word.

Lns 140–141: the sentence seems to be redundant with the following one

Ln 160: change "no" to "hardly any"

Ln 180: change "the most parsimonious" to "one" – I am not sure how much more parsimonious is this hypothesis over any other.

Ln 195: consider deleting "reverse-transcribing".

Ln 198: virtual reality is not a reality; is virtual extinction an extinction?

Lns 206–207. More potent than what. And what is "more sophisticated"?

For Discussion: badnaviruses are pararetroviruses, and yet they infect trees and shrubs (cacao, raspberry, spirea...).

*Authors' response:*

We have accommodated virtually (meaning 'nearly') all editorial changes proposed by Dr. Mushegian.

He has also raised an important discussion point: if, according to our hypothesis, woody plants such as grapevine evolved resistance to pararetroviruses via exaptation of pararetroviral inserts, why do fully infectious pararetroviruses occur at least in some species of the woody plants? Certainly, this implies that our hypothesis is not universally applicable to all woody plants. This, however, is hardly a surprise given that 'woodiness' has evolved independently in many families of the gymnosperms and angiosperms that likely had varying initial levels of both the exposure and resistance to diverse virus lineages. It is also possible that the early domestication and vegetative propagation of the grapevine have increased the virus pressure and accelerated emergence of a resistance to pararetroviruses.

### Reviewer's report 2

Dr. I. King Jordan (Georgia Institute of Technology, Atlanta, GA, USA)

*Reviewer comments:*

The authors of this paper report the discovery of partial reverse transcriptase encoding open reading frames, of apparent pararetroviral origins, in two grapevine genomes. The authors also demonstrate that several previous claims for the positive strand RNA viral origins of grapevine genome sequences actually represent experimental and/or annotation artifacts. The discovery of pararetroviral genomes is interesting since grapevine plants do not appear to be subject to infection by pararetroviruses, unlike numerous herbaceous plants. The authors hypothesize that the pararetroviral sequence inserts reported here confer immunity to pararetrovirus infection via an RNA interference (RNAi) like mechanism based on stochastic transcription of the integrated viral sequences.

This is an intriguing hypothesis and the authors lay out two lines of research that can be used to test their idea: 1) a census of all grapevine viruses using metagenomics and 2) an investigation of the mechanisms of RNAi in woody plants. I would like to suggest two other tests of their hypothesis, which while less direct may be easier to carry out.

First, it would be interesting to know if these pararetroviral sequence inserts actually encode small RNAs and/or if they are expressed as RNA at all. This could be addressed computationally as with the work presented here. For instance, are there small RNA libraries for grapevine that could be queried? Are there ESTs that support the expression of these pararetroviral like inserts? This could also be assessed experimentally with RT-PCR for example.

Second, the authors suggest that the presence of pararetroviral sequences in the grapevine genomes is consistent with the idea that 'heritable maintenance of pararetroviral sequences has potential benefits for the host plants.' If this is indeed the case, then one may expect that the pararetroviral sequence inserts are conserved over evolutionary time. As with the expression of pararetroviral sequences, this point could be addressed computationally and/or experimentally. Two genome sequences are analyzed here but it is unclear if the pararetroviral sequences discovered are conserved at orthologous positions in the two genomes. If so, are these sequences conserved more or less than protein coding gene sequences between the plants? A PCR survey of multiple Vitaceae related strains and species could be conducted to look for orthologous pararetroviral sequences as was done for the previously misidentified GLRaV-8 sequences.

The defense hypothesis would seem to suggest that the pararetroviral sequence inserts uncovered here are still effective at guarding against infection by pararetroviruses. Yet the authors propose that the pararetroviral sequences in the grapevine are "derived from the currently extinct, grapevine-specific pararetroviruses." Given the need for sequence identity between small RNAs and RNA/DNA targets in RNAi systems, how is it that these ancient inserts could still be effective at maintaining immunity against pararetrovirus insertions?

Integrated pararetrovirus sequences are also found in the genomes of herbaceous plants, but herbaceous plants are susceptible to pararetrovirus infection. The authors propose that long-lived woody plants, such as grapevine, evolved more vigorous RNAi defense mechanisms than more short lived herbaceous plants. Is it known that herbaceous plants mount less effective responses to foreign agents in general? Are there any other lines of evidence or references in support of this idea?

From a technical perspective, it would help to have a bit more detail on the methods of sequence analysis used here. It is not possible to understand what kind of analysis was conducted based on the information provided. For instance, what program was used to compare sequences? Were comparisons done using nucleotide (as stated) or protein (as implied) sequences or both? In addition, the authors refer to "highly significant similarity" between grapevine genome sequences and pararetroviruses but no statistics are shown.

Minor point: It would be helpful if the URL listed for the Grape Genome Browser pointed straight the browser  as opposed to the front page of Genoscope.

*Authors' response:*

We very much appreciate Dr. Jordan's idea to investigate possible transcription of the pararetroviral inserts in grapevine into siRNAs or other small RNAs that may enable RNAi antipararetroviral response either computationally or experimentally. Although we are not aware of the grapevine small RNA or EST databases, deep sequencing of the grapevine transcriptome in general and small RNAs in particular would be certain to generate required data. Similarly, testing conservation of the pararetroviral inserts in different grapevine varieties and species is a promising idea that can reveal when the events of virus sequence insertions has occurred relative to diversification of the family *Vitaceae *or genus *Vitis*. In fact, this will be more doable when the several homozygous grapevine genomes are available. The existing genome sequences of two cultured variants of Pinot Noir are more problematic to compare because one of these is highly homozygous whereas another is highly heterozygous.

The question of how pararetroviral inserts may still be effective against the challenge of extant viruses if they are derived from now extinct viruses is very intriguing. One possible scenario is that there are no pararetroviruses left that are capable of infecting grapevine. This would be analogous to extintion of the smallpox virus due to the global vaccination program. If this were true, the pararetroviral inserts themselves could have lost their selective advantage and will gradually deteriorate. An alternative scenario is that the grapevine-specific pararetroviruses closely related to the viral inserts found in grapevine genome still lurk in the wild plant host species. If this were the case, gradual sequence evolution of such viruses could result in eventual escape from the antiviral control mediated by the existing inserts. The census of extant pararetroviruses, as well as investigation of viral insert-derived small RNAs discussed above could help to distinguish between these scenarios.

The next question is concerned with the evidence for more vigorous antiviral defenses in woody versus herbaceous plants. To the best of our knowledge this remains just a plausible hypothesis. The only circumstantial supporting evidence we are aware of is that many RNA viruses infecting woody or perennial plants, but not those infecting annual herbaceous plants have acquired an AlkB domain apparently involved in repairing the methylation damage to the viral RNA (ref. [31]). This observation can be interpreted to suggest that woody plants have evolved an additional line of antiviral defense via targeted methylation of the viral RNAs. This possibility, as well as better understanding of RNAi machinery in woody plants are very promising directions for the future research.

We have also made several modifications to account for the raised technical issues.

### Reviewer's report 3

Dr. Eugene V. Koonin (National Institutes of Health, Bethesda, MD, USA)

*Reviewer comments:*

In this interesting Hypothesis paper Bertsch et al demonstrate the presence in the grapevine genome of multiple sequence segments homologous to parts of pararetrovirus genomes and hypothesize that these integrated virus-derived sequences confer resistance to the respective viruses via an RNAi mechanism. The observation is not entirely novel because integrated pararetrovirus sequences have been reported previously in other plant genomes (petunia and rice) as duly cited in this manuscript. There is, however, a very interesting new point here, namely, that those other, herbaceous plants are susceptible to pararetroviruses, whereas no pararetroviruses of grapevine are known in spite of exhaustive virological study of this plant. So the authors reasonably conjecture that the RNAi-based defense mechanisms are particularly important and hence especially efficient in a woody plant like grapevine. In addition to these findings and ideas, this work puts to rest the previous erroneous reports on the presence, in the grapevine genome, of segments homologous to certain positive-strand RNA viruses. Unlike pararetroviruses, positive-strand RNA viruses have no reverse transcription step in their reproduction, so the possibility of integration is dubious – and would be a sensation of sorts if confirmed. I think it is important to dissuade such myths.

This is not an earth-shattering discovery but the paper is interesting, has valuable biological implications, and will attract the reader's attention to a remarkable phenomenon. I would like to make two comments, one of a fundamental nature, the other one more on the technical/presentational side.

1. The authors suggest that there are no extant pararetroviruses of grapevine and moreover that the virus-derived sequences in the grapevine genome represent extinct viruses. I think one should be more cautious on these issues because known RNAi-based antiviral defense mechanisms require exact complementarity between the siRNA and the target. So if those target viruses are indeed extinct, that extinction must have been a recent event. Else, the viruses still might be around but cannot replicate in grapevine owing to the interference from the endogenous homologous sequences. This deserves a more careful and nuanced discussion. Along more or less related lines, I would be quite interested to know whether or not the viral inserts are homologous in other grapevine varietals ? is there any chance to find out ?

2. I am not sure the current manuscript is documented/illustrated as fully as possible. Table 1 is a rather sketchy characterization of the viral-like sequences in the grape genome. I would rather see at least a couple of alignments, and perhaps, a schematic showing the chromosomal location of these sequences. Is there anything unusual in their genomic surroundings ? Any ideas on how they could be expressed ?

*Authors' response:*

We are grateful to Dr. Koonin for his incisive comments. First of these comments resonates with one by Dr. Jordan. Indeed, it is perfectly feasible that pararetroviruses that were formerly capable of infecting grapevine are currently surviving in different host plants. It will also be important to learn how conserved are the pararetroviral inserts in a wide variety of grapevine cultivars, and if and how they are expressed. However, we agree that both the acquisition of pararetroviral inserts by grapevine and the apparent extinction of the parental pararetroviruses could be the relatively recent events, perhaps associated with the domestication of grapevine in the Caucasus 8–10 thousand years ago.

As requested, we have included a multiple alignment of the conserved regions of the pararetroviral inserts with those in the most closely related infectious pararetroviruses (Fig. 1) and a chromosomal map showing location of the inserts.

## Supplementary Material

Additional file 1 **Figure S1**. Positions of the pararetrovirus-related inserts in the grapevine chromosomes. Click here for file
