# Supplementary material for: Retention of the virus-derived sequences in the nuclear genome of grapevine as a potential pathway to virus resistance
Source: Biol Direct. 2009 Jun 26;4:21. doi: 10.1186/1745-6150-4-21 (PMC2714080; doi:10.1186/1745-6150-4-21)
Supplement: Additional file 1 — Figure S1. Positions of the pararetrovirus-related inserts in the grapevine chromosomes. [file 1745-6150-4-21-S1.ppt]

## Slide 1
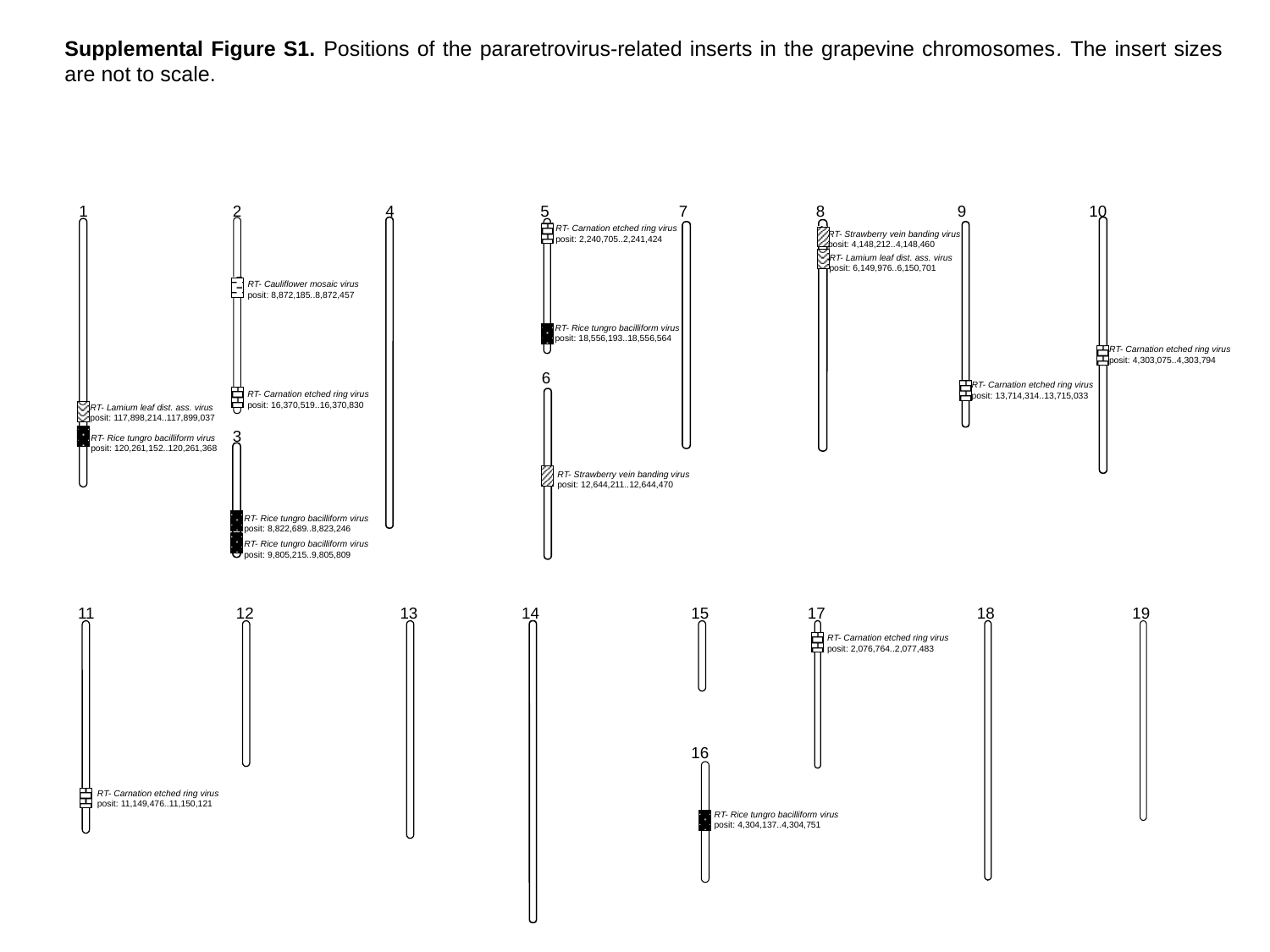

Supplemental Figure S1. Positions of the pararetrovirus-related inserts in the grapevine chromosomes. The insert sizes are not to scale.
1
2
4
5
7
8
9
10
RT- Carnation etched ring virus
posit: 2,240,705..2,241,424
RT- Strawberry vein banding virus
posit: 4,148,212..4,148,460
RT- Lamium leaf dist. ass. virus
posit: 6,149,976..6,150,701
RT- Cauliflower mosaic virus
posit: 8,872,185..8,872,457
RT- Rice tungro bacilliform virus
posit: 18,556,193..18,556,564
RT- Carnation etched ring virus
posit: 4,303,075..4,303,794
6
RT- Carnation etched ring virus
posit: 13,714,314..13,715,033
RT- Carnation etched ring virus
posit: 16,370,519..16,370,830
RT- Lamium leaf dist. ass. virus
posit: 117,898,214..117,899,037
RT- Rice tungro bacilliform virus
posit: 120,261,152..120,261,368
3
RT- Strawberry vein banding virus
posit: 12,644,211..12,644,470
RT- Rice tungro bacilliform virus
posit: 8,822,689..8,823,246
RT- Rice tungro bacilliform virus
posit: 9,805,215..9,805,809
11
12
13
14
15
17
18
19
RT- Carnation etched ring virus
posit: 2,076,764..2,077,483
16
RT- Carnation etched ring virus
posit: 11,149,476..11,150,121
RT- Rice tungro bacilliform virus
posit: 4,304,137..4,304,751
